# Multicenter Study of Buruli Ulcer Disabilities in the Head and Neck Region

**DOI:** 10.1155/2011/647418

**Published:** 2011-04-26

**Authors:** Pius Agbenorku

**Affiliations:** Reconstructive Plastic Surgery and Burns Unit, Komfo Anokye Teaching Hospital, School of Medical Sciences, Kwame Nkrumah University of Science and Technology, University Post Office, KNUST, Kumasi, GH, Ghana

## Abstract

*Objective*. To identify disabilities caused by Buruli Ulcer Disease (BUD) when it affects the Head and Neck Region (HNR) of patients in endemic areas and suggest possible ways to overcome the complications involved. *Methods*. Data for the study was collected from six different hospitals in the central part of Ghana from 2004–2009. Diagnosis of BUD was based on clinical findings and confirmed by positive result of Ziehl-Neelson Test for Acid Fast Bacilli, Polymerase Chain Reaction, or Histopathology. Treatment of BUD involved a combination of surgical interventions and antimycobacterial chemotherapy for 8 weeks. *Results*. The age of the 38 patients ranged from 0–56 years (mean age of 14.3 years), with males outnumbering females. Most (55.3%, *N* = 21) of the patients reported to the facilities with developed BUD deformities. Patients who lost their eyeball (*N* = 5) recorded the highest in terms of functional disability. A mean total hospital stay of 52 days and follow-up period of 2.3 years were recorded for the study. *Conclusion*. Visual impairment was the commonest form of disability recorded in the HNR. Management difficulties and BUD disabilities could be avoided by early detection of the disease and training of health professionals at district levels.

## 1. Introduction

Buruli ulcer (BU) is a severe disabling and disfiguring disease caused by *Mycobacterium ulcerans* (MU). Even though cases are reported in all age groups, it affects primarily children less than 15 years of age [1, 2]. Oluwasanmi et al. [3] and van der Werf et al. [2] did not find any sex difference in their studies, but Barker reported prevalence to be higher among women than men and among boys than girls. One characteristic of the disease is its apparent association with aquatic habitat, especially in many tropical and subtropical countries [3–6]. In early or preulcerative lesions, MU produces a lipid toxin, mycolactone, which is responsible for necrosis of the dermis, panniculus, and fascia, culminating in extensive ulcers [7]. Preulcerative lesions and small ulcers may be surgically excised and closed. However, antimicrobial drug therapy is often effective for treatment of early lesions [8]. Large ulcers generally require excision followed by skin grafting [9] in combination with BU chemotherapy [10].

In Ghana, BU is currently the second mycobacterial infection after tuberculosis [11]. According to the Ghana National Buruli Ulcer Control Programme, January—December 2009 Report, 19 cases of BU in the Head and Neck Region (HNR) had been confirmed in various regions of the country, with Ashanti Region (in Central Ghana) alone recording 52.6% of it [12]. BU may affect all parts of the body but most frequently the lower extremities. The disease starts as a painless nodule or papule in the skin. If this is not excised, necrosis of the skin and subcutaneous tissue may result [13–15]. Sometimes it may cause tendinous or bone exposure; when it involves a joint, contracture may result. MU disease in the HNR, especially the face, may lead to serious sequelae such as ulcerative destruction of the eyelids, loss of the nose, or deformity of the face [16, 17]. Agbenorku in 2005 reported BU in the HNR in his study conducted in Ghana [17]. Similarly, Kouame et al. in Cote D'Ivoire [18] and Phanzu et al. in Angola [19] had also reported BU in the HNR.

*M. ulcerans* disease of the scalp may also lead to permanent alopecia. After treatment of the ulcer, complex reconstructive procedures may be required to restore a more or less desired hairstyle. The objective of this retrospective study was to identify disabilities caused by Buruli Ulcer Disease (BUD), when it affects the HNR of patients in endemic areas and suggests possible ways to overcome the complications involved.

## 2. Patients and Methods

### 2.1. Data Acquisition

Data for the study was retrieved from six different hospitals, which are: Komfo Anokye Teaching Hospital, Kumasi; Global Evangelical Mission Hospital, Apromase; SDA Hospital, Asamang; Bekwai District Hospital, Bekwai; Nyinahin Government Hospital, Nyinahin and Nkawie District Hospital, Nkawie all in the middle belt of Ghana, from 2004–2009. The sources of information were the operation registers and the case notes of the patients. Information obtained included age, sex distribution, clinical presentation (preulcerative and ulcer), location of BUD, deformities presented, and surgical and antibiotic therapy. Preulcerative lesions include plaque, papule and nonulcerative edematous forms. Ethical clearance for the study was obtained from the Ashanti Regional Health Directorate. Data obtained was recorded and displayed in tables and graphs by using SPSS version 16.0 (SPSS, Inc., Chicago, Ill, USA).

### 2.2. Clinical and Laboratory Diagnosis

Diagnosis of BU was based on clinical findings such as chronicity of the wound, typical undermined edges with central necrotic tissues, and failure to respond to traditional wound management procedures and antibiotic therapy. The findings were then confirmed by any two of the following laboratory diagnosis: Ziehl-Neelson Test for Acid Fast Bacilli, Polymerase Chain Reaction, and Histopathology.

### 2.3. Patients Management

#### 2.3.1. Patients Admitted with Deformities

The patients had surgical excisions of the chronic ulcers. Some of them, because of the location of the ulcers (immediately adjacent to the eye) could not have complete excision. They were dressed over long periods with normal saline or 2% acetic acid lotions. Because of the difficulty in achieving good hemostasis, some of the excised ulcers were grafted secondarily after 24–72 hours. Ulceration that extended to the eyelid and base of the nose were treated by four surgical excisions, daily wound dressings, and skin grafts. Patients with eyelid destruction had them reconstructed with an Indian forehead flap. Surgery was combined with antimycobacterial chemotherapy (Rifampin and Streptomycin) for 8 weeks. The treatment necessitates long hospitalization and is quite expensive with less complications. The patients came for followup monthly for three visits and then quarterly.

#### 2.3.2. Patients without Deformities

Patients with BU that had good healthy edges with hypertrophic granulation had only sharp debridement done. After meticulous hemostasis, the wounds were covered with split-thickness skin grafts or local transposition flaps. The grafts were dressed with Vaseline gauze and tie-over dressings. The recipient site dressings were changed on the 4th or 5th postoperative day while the donor sites (normally the thigh) were changed on the 14th day after operative day. Similarly, surgery was combined with Rifampin and Streptomycin treatment for 8 weeks. Most of the grafts healed within two weeks and the patients were discharged home.

## 3. Results

### 3.1. Demographic Characteristics of Patients

**Figure 1:**
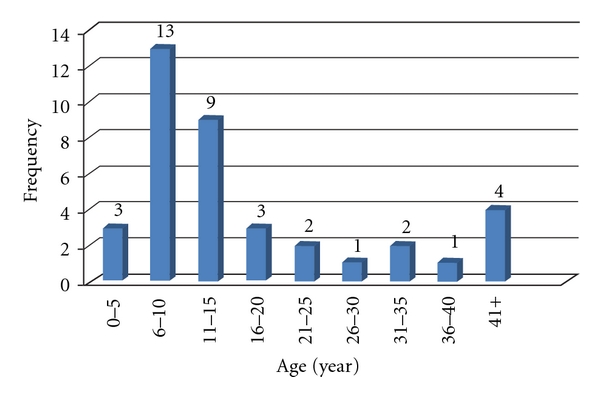
Age distribution of patients with BU in the HNR.

During the study period, 38 patients with BU in the HNR were recorded of which males' outnumbered females of a ratio 3 : 2, respectively. The age of patients ranged from 0–56 years (mean age of 14.3 years), while majority (65.8%, *N* = 25) of them were pupils (5–15 years), as shown in Figure 1. In addition, total hospital stay of patients ranged from 15–60 days (Mean duration of 52 days), while follow-up period of the study ranged from 1–5 years (Mean duration of 2.3 years), of which majority (85%, *N* = 32) of the patients were captured in the period. Again, most (55.3%, *N* = 21) of the patients reported to the facilities with already developed BUD deformities. More than half (60.5%, *N* = 23)) of the patients developed facial scars after treatment.

### 3.2. Clinical Stages and Location

**Figure 2:**
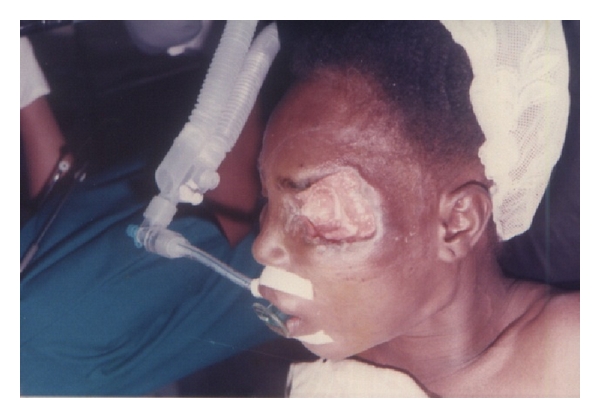
Patient with BUD on the eye region.

**Figure 3:**
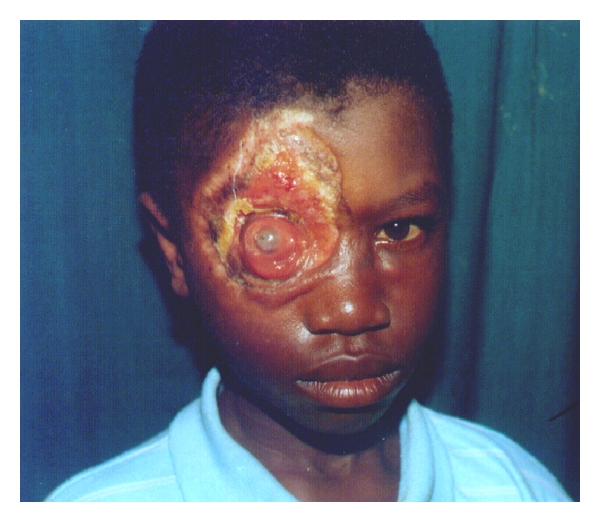
Patient with destroyed Eyeball due to BUD.

**Figure 4:**
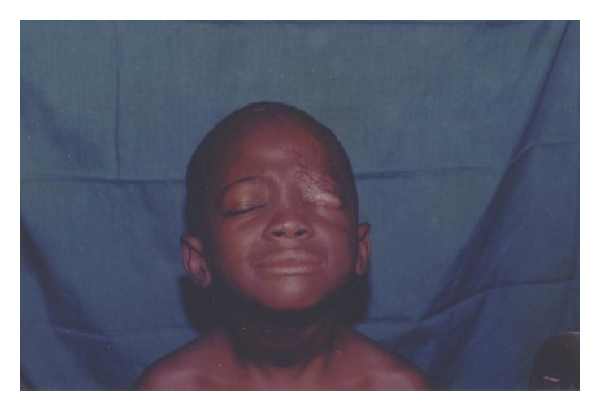
Patient with BUD affecting the Eyelid.

**Figure 5:**
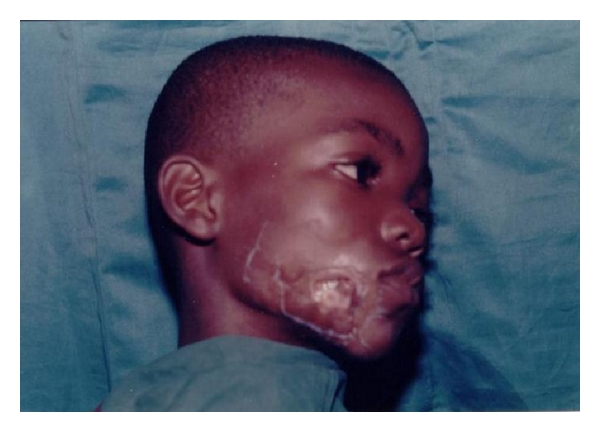
Patient with BUD located on the cheek.

**Figure 6:**
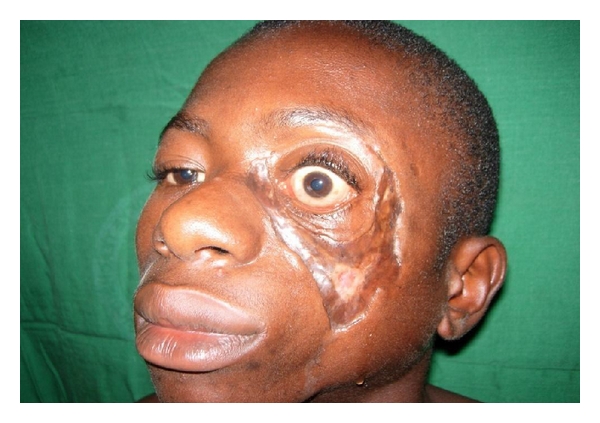
Facial Scar developed after Initial Surgery of a BU Patient.

The most prevalent clinical presentations of the disease in the HNR were severe and mild ulcers (76.3%, *N* = 29), while no nodular stage was recorded. In terms of location BUD on the HNR, three divisions (Eye & Forehead; Cheek & Nose; Neck regions) were made, with, the Eye and Forehead region, recording the highest (59.6%, *N* = 23) (Table 1). Representative results of the locations are shown in Figures 2, 3, 4, 5, 6.

### 3.3. BUD Deformities Presented by Patients

A high number (55.3%, *N* = 21) of the BU patients reported to the facilities with already developed deformities. These sequelae includes stiffness of the neck (*N* = 4), loss of part of the ear (*N* = 2), loss of part the nose (*N* = 1), loss of part of the lower lips (*N* = 2), loss of the eyeball (*N* = 5), loss of the eyelid (*N* = 4), and baldness (*N* = 3) at the frontal part of the head.

## 4. Discussion

Infection due to *Mycobacterium ulcerans* or BU usually occurs on the limbs or trunk. Involvement of the head and neck region is less frequent but can lead to serious sequelae. Novelties in the study includes BU treatment regime involving the HNR of patients with or without sequelae and data to support the fact that BUD is also detrimental not only the limbs of the body, but also the HNR. According to the World Health Organisation (WHO), a functional concept of disability, defines a disability as any long-term limitation in activity resulting from a condition or health problem.

The study confirms findings elsewhere that the disease affects children more than adults [1, 2]. Marston et al. found the highest rate of infection among children 10–14 years of age in a disease-endemic area in the Daloa Region of Côte d'Ivoire [20]. This observation has been misinterpreted to mean that the disease affects only children. This study demonstrates that all age groups can be affected; however, children (6–10 years) recorded the highest infection rate in terms of BUD in the HNR. The results of our study again reveal that males outnumbered females and this had been confirmed by many BU studies [11, 17, 18]. 

In the “BU on the face” case report of Phanzu et al., series of surgical interventions were performed and antimycobacterial chemotherapy (Rifampin and Ciprofloxacin) was given for sixty days [19]. Similar surgical interventions were performed in this study especially, for patients who delayed in seeking medical treatment (i.e., patients who reported with BU deformities), however, a different set of antimycobacterial chemotherapy was used in this study. The longer stay of patients in the hospital was also ascertained by the study, especially for patients who reported with BU deformities, since the mean hospital duration was fifty-two days. These cases demonstrate the management difficulties of BU on the face and suggest that cases of these sorts should be managed judiciously by professionals in the field to enhance healing. 

The study has shown that BUD in central Ghana is much more widespread than previously thought. In all areas where BU cases have been identified, the extent of the disease is likely to be much greater than currently recognized through the routine reporting system. According to the Ghana National BU Program Report of 2009, ten HNR BU cases were recorded from Agogo Presbyterian Hospital (Asante Akim North District) and SDA Hospital at Asamang, Sekyere South (formally Afigya Sekyere) District [12]. This study confirms that in Central Ghana part of the distribution pattern of the disease is in the Ashanti Region; this is very essential, since it may serve as a guide to other studies and also serve as a tool to assess the disability impact of BUD, taking into consideration the HNR of the patients.

The BUD usually starts as a nodule in the skin. If the nodule is excised, the extensive ulceration can be prevented [15, 16]. Most of these patients reported very late for treatment, some even compounded it by using herbal medications, which were additional source of secondary microorganism infections. The 2009 Ghana National BU Report indicates that, 64.2% (*N* = 508) of the clinical presentations were ulcers [12], and this had also been confirmed by other BU studies [10, 11, 15, 21]. As revealed in Table 1 of this study, most of the clinical presentations recorded were ulcers affecting almost every part of the HNR of the patients. The result of the study was in line with the already published study; majority of the patients who reported to the various facilities with ulcers were those with deformities that led to functional disabilities such as blindness which affects patients in their daily activities. Facial disfigurement as a result of the facial scars due to the surgeries also caused psychological effects (e.g., low self-esteem, shyness, and inferiority complex), which could affect the victims in their education (especially in children), marriage, and work. 

BUD on the face in particular posed a series of challenges to the plastic surgeon. In the first place, unlike BUD on other parts of the body where the surgeon could eventually excise all the diseased lesions, this is often not possible in the case of facial BU. The essential organs, especially the eyes, limit the surgeon who indeed would try to prevent any iatrogenic injury to the eyes. The leftover diseased tissues could eventually infiltrate the eyes which could lead to their blindness. In this study, all patients with loss of eyeball reported to the facility as such, a contributing factor to the loss of sight is the fact that all patients reported very late for treatment. Public health education in BUD endemic communities should be intensified. This will prompt patients to report early enough for diagnosis and treatment.

Equally important is the management of the disease in the district hospitals. Most, district and community health institutions have no “tools” for diagnosis and treatment of BUD. Equipping and training of health personnel in the district hospitals is vital, since these facilities are always the first place of visit by most patients. The existing BU management team, especially in endemic districts should be provided with all necessary “tools” to facilitate their work. Establishing surveillance teams in the districts should be a necessity, for rapid diagnoses of BUD in order to prevent disabilities. In situations where patients for some reasons report very late with advanced ulcers, they should be referred immediately to tertiary centers to be managed by specialist.

## 5. Conclusion

The study demonstrated that visual impairment was the commonest form of disability in the HNR. Facial scars caused by BUD also could have a negative impact on the patients, self-confident. Thus, management difficulties and disabilities caused by BUD in the HNR of patients could be avoided by early detection, treatment, and health education on the disease. The outcome in these patients also underscores the importance of prompt referral of suspected cases and training of health professionals at the district levels in the early diagnosis of BU.

## Figures and Tables

**Table 1:** Clinical presentation and location of BU in the HNR.

Location	Clinical presentation				
	Severe ulcer	Mild ulcer	Plaque	Edematous forms	Total (%)
Eye & forehead region	8	12	2	1	23 (59.6)
Cheek & nose region	2	0	0	2	4 (10.5)
Neck region	3	4	3	1	11 (29.9)

Total (%)	13 (34.2)	16 (42.1)	5 (13.2)	4 (10.5)	

## References

[1] Uganda Buruli Group (1969). BCG vaccination against *Mycobacterium ulcerans* infection (Buruli ulcer). *Lancet*.

[2] van der Werf TS, van der Graaf WTA, Groothuis DG, Knell AJ (1989). *Mycobacterium ulcerans* infection in Ashanti region, Ghana. *Transactions of the Royal Society of Tropical Medicine and Hygiene*.

[3] Oluwasanmi JO, Solanke TF, Olurin EO (1976). *Mycobacterium ulcerans* (Buruli) skin ulceration in Nigeria. *American Journal of Tropical Medicine and Hygiene*.

[4] Muelder K (1992). Wounds that will not heal. *International Journal of Dermatology*.

[5] Portaels F, Johnson P, Meyers WM (2001). *Buruli ulcer. Diagnosis of Mycobacterium ulcerans Disease*.

[6] Janssens P, Pattyn S, Meyers WM, Portaels F (2005). Buruli ulcer: a historical overview with updating to 2005. *Bulletin des Seances/Academie Royale des Sciences d'Outre-Mer*.

[7] Asiedu K, Sherpbier R, Raviglione M (2001). *Buruli ulcer—Mycobacterium ulcerans Infection*.

[8] Etuaful S, Carbonnelle B, Grosset J (2005). Efficacy of the combination rifampin-streptomycin in preventing growth of *Mycobacterium ulcerans* in early lesions of Buruli ulcer in humans. *Antimicrobial Agents and Chemotherapy*.

[9] Sizaire V, Nackers F, Comte E, Portaels F (2006). *Mycobacterium ulcerans* infection: control, diagnosis, and treatment. *Lancet Infectious Diseases*.

[10] Agbenorku P, Agbenorku M, Saunderson P, Lehman L (2006). The benefits of a combination of surgery and chemotherapy in the management of Buruli ulcer patients. *Journal of Science and Technology*.

[11] Amofah G, Bonsu F, Tetteh C (2002). Buruli ulcer in Ghana: results of a national case search. *Emerging Infectious Diseases*.

[12] http://www.burulighana.org/files/SummaryNat_Data.pdf.

[13] Agbenorku P (2001). Hypertrophic scars. *Buruli Ulcer. Diagnosis of *Mycobacterium ulcerans* Disease. A Manual for Health Care Providers*.

[14] Leonardo J (2000). PSEF International Scholar sees allies in Buruli ulcer battle. *Plastic Surgery News*.

[15] Agbenorku P, Kporku H (2001). Buruli ulcer: a poverty disease?. *Indian Journal of Clinical Practice*.

[16] Agbenorku P (2000). *Mycobacterium ulcerans* skin ulcer (MUSU) of the face. *West African Journal of Medicine*.

[17] Agbenorku P (2005). *Mycobacterium ulcerans* disease in the head and neck region. *Indian Journal of Clinical Practice*.

[18] Kouame K, Ecra E, Cisse M (2008). Buruli ulcer involving the head: outcomes and therapeutic aspects in 8 cases observed at the University Hospital Center in Abidjan, Cote d'Ivoire. *Medecine Tropicale*.

[19] Phanzu DM, Ablordey A, Imposo DB (2007). Short report: edematous *Mycobacterium ulcerans* infection (Buruli ulcer) on the face: a case report. *American Journal of Tropical Medicine and Hygiene*.

[20] Marston BJ, Diallo MO, Horsburgh CR (1995). Emergence of Buruli ulcer disease in the Daloa region of Côte d’Ivoire. *American Journal of Tropical Medicine and Hygiene*.

[21] Agbenorku P, Akpaloo J, Amofa GK (2000). Sequelae of *Mycobacterium ulcerans* infections (Buruli ulcer). *European Journal of Plastic Surgery*.

